# Learning from scaling up ultra-rapid genomic testing for critically ill children to a national level

**DOI:** 10.1038/s41525-020-00168-3

**Published:** 2021-01-28

**Authors:** Stephanie Best, Helen Brown, Sebastian Lunke, Chirag Patel, Jason Pinner, Christopher P. Barnett, Meredith Wilson, Sarah A. Sandaradura, Belinda McClaren, Gemma R. Brett, Jeffrey Braithwaite, Zornitza Stark

**Affiliations:** 1 Australian Institute of Health Innovation, Macquarie University, Sydney, NSW Australia; 2 Australian Genomics, Melbourne, VIC Australia; 3 Faculty of Health Science, Deakin University, Melbourne, VIC Australia; 4 Victorian Clinical Genetics Services, Murdoch Children’s Research Institute, Melbourne, VIC Australia; 5 The University of Melbourne, Melbourne, VIC Australia; 6 Genetic Health Queensland, Royal Brisbane and Women’s Hospital, Brisbane, QLD Australia; 7 Sydney Children’s Hospitals Network - Randwick, Sydney, NSW Australia; 8 Paediatric and Reproductive Genetics Unit, South Australian Clinical Genetics Service, Adelaide, SA Australia; 9 Sydney Children’s Hospitals Network - Westmead, Sydney, NSW Australia; 10 Discipline of Genomic Medicine, University of Sydney, Sydney, NSW Australia; 11 Discipline of Child and Adolescent Health, University of Sydney, Sydney, NSW Australia

**Keywords:** Health care, Health services

## Abstract

In scaling up an ultra-rapid genomics program, we used implementation science principles to design and investigate influences on implementation and identify strategies required for sustainable “real-world” services. Interviews with key professionals revealed the importance of networks and relationship building, leadership, culture, and the relative advantage afforded by ultra-rapid genomics in the care of critically ill children. Although clinical geneticists focused on intervention characteristics and the fit with patient-centered care, intensivists emphasized the importance of access to knowledge, in particular from clinical geneticists. The relative advantage of ultra-rapid genomics and trust in consistent and transparent delivery were significant in creating engagement at initial implementation, with appropriate resourcing highlighted as important for longer term sustainability of implementation. Our findings demonstrate where common approaches can be used and, significantly, where there is a need to tailor support by professional role and implementation phase, to maximize the potential of ultra-rapid genomic testing to improve patient care.

## Introduction

Rapid genomic diagnosis can influence the management of critically ill infants and children with genetic conditions^1^. Despite growing evidence of efficacy, implementation to date has been mostly limited to single academic centers, and most of the reported outcomes have been restricted to diagnostic yield and clinical utility^2–6^. Although it is recognized that implementation science principles can be used to cultivate a learning healthcare system^7^, expediate spread and scaling up of innovation and provide a framework for iterative learning, aiding replication, and planning^8,9^, there are concerns that the limited application of such frameworks is hampering efforts to integrate genomics into healthcare^10^. To overcome this impasse, effectiveness-implementation hybrid study designs^11^ can be used to evaluate processes and outcomes simultaneously, thereby reducing time to translation of findings into clinical practice. Employing a hybrid design from the outset allows a structured approach to early identification of implementation strategies to match the specific clinical context^12,13^, while the evidence base for clinical efficacy continues to grow.

**Fig. 1:**
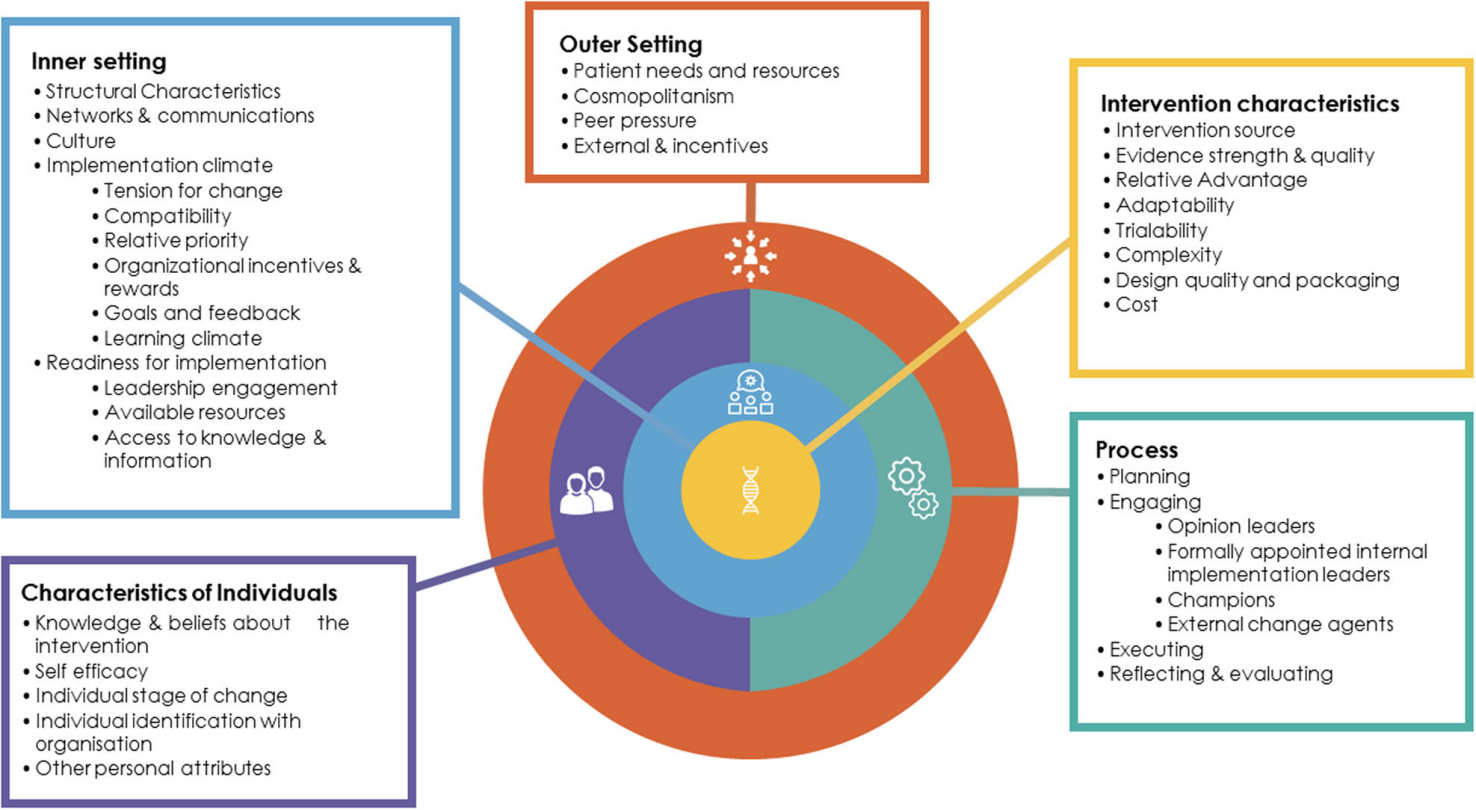
Consolidated Framework for Implementation Research (CFIR)^14^. Lists of the five domains (Inner setting, outer setting, intervention characteristics, process and characteristics of individuals) and underlying constructs.

Such hybrid study designs may be used alongside frameworks such as the Consolidated Framework for Implementation Research (CFIR)^14^, which facilitates systematized understanding of the influences on the implementation process and can be used to inform implementation strategies. Formed from five domains with 39 underlying constructs (Fig. 1), the CFIR covers: (i) Intervention characteristics, e.g., evidence strength and quality; (ii) Outer setting, e.g., economic, political and social context; (iii) Inner setting, e.g., culture and context of the organization; (iv) Characteristics of individuals, e.g., personal traits and belief about capabilities; (v) Process, e.g., planning and reflection^14^. The CFIR has been used in the genomics context^15^ to describe contextual factors influencing uptake, for example, identifying key drivers needed for sustainable clinical genomic programs^16^, system-level barriers^17^ and challenges and strategies for implementation^18^. However, there is limited application of implementation science findings into “real life” adoption of clinical genomics in practice.

We used the CFIR to design a collaborative multi-site network, the Australian Genomics Acute Care program, to deliver ultra-rapid genomic testing across 12 tertiary hospitals and two laboratories in Australia^19^. This highly coordinated clinical and laboratory program in a public healthcare system delivered laboratory reports with a mean time of 3.3 calendar days (95% CI: 3.2–3.5), with a diagnostic yield of 51%. A high degree of consistency was achieved between the clinical and laboratory sites, with little variability in the duration of each step of the diagnostic pathway (time from hospital admission to test initiation, consent, sample transport, or reporting). However, there was marked variation in rates of recruitment between sites, and the longest component of the diagnostic pathway remained time from hospital admission to test initiation, resulting in an overall time from hospital admission to genomic result of 17.5 days (95% CI 14.6–21.1)^19^.

Here, we sought to apply implementation science principles and theory to investigate the experience of key professionals involved in delivering the Australian Genomics Acute Care program across multiple sites, in order to examine major influences on implementation and identify future implementation strategies for sustainable ultra-rapid genomics services.

### Research context and design

Healthcare in Australia is funded through a combination of public and private sources; about a third of services are private, and two-thirds public. The federal government is responsible for the universal public health insurance scheme and both the state and federal governments fund the local public hospitals and associated services. Clinical genomic testing in Australia is currently funded by the federal^20^ and state governments^21^ through a mixture of healthcare funding and investments in major translational genomics projects such as the Australian Genomics Health Alliance.

**Fig. 2:**
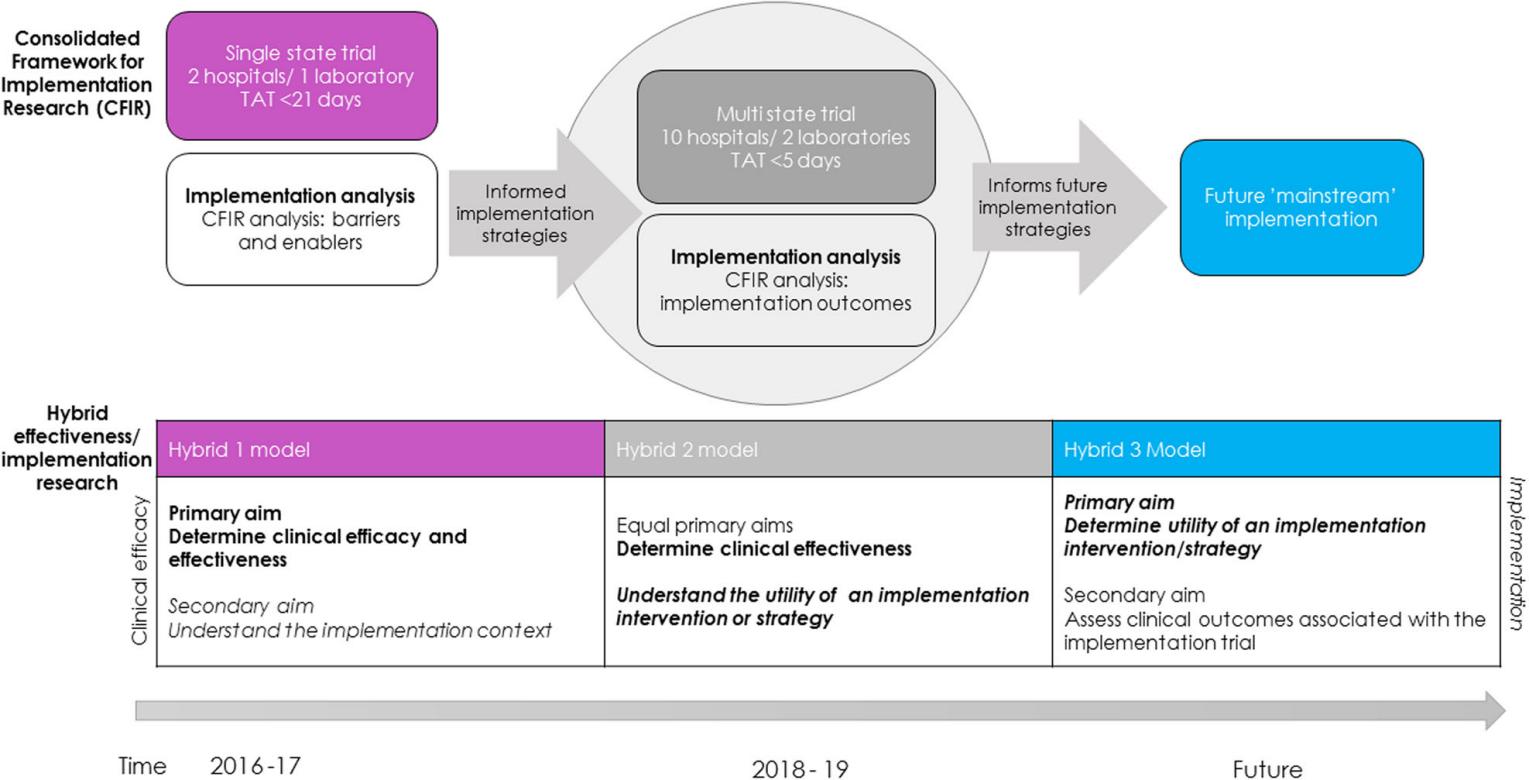
Applying an Effectiveness-Implementation Hybrid study design^11^ to evaluate clinical and implementation aims. This timeline demonstrates the use of the Consolidated Framework for Implementation Research (CFIR) and the hybrid effectiveness/implementation model as the programme progresses. Initially, as a hybrid 1 effectiveness/implementation model, the single state trial in 2016-7 placed priority on producing evidence on clinical effectiveness. As clinical effectiveness evidence developed, the multistate trial (the focus of this paper) in 2018-9 ran as a hybrid model 2, with equal focus on generating implementation and clinical effectiveness evidence. Future programmes will be hybrid model 3 with attention on creating evidence to support implementation.

The Australian Genomics Acute Care program built upon the prior experience of implementing rapid genomic testing (time to result <21 days) across two hospitals in 2016–17, as part of a Hybrid 1 effectiveness-implementation study (Fig. 2) while clinical effectiveness was in the early stages of being established^22^. Leveraging research and operational infrastructure provided by the Australian Genomics Health Alliance, a national genomic medicine initiative^20^, the Acute Care program scaled up ultra-rapid genomic testing to multiple sites using a Hybrid 2^11,23^ effectiveness–implementation study design during 2018–19, as evidence of clinical effectiveness increased^1,2,6^. We used the CFIR to design an implementation plan emphasizing communication and feedback, standardized processes, co-ordination, distributed leadership, and collective learning^19^. This Hybrid 2 study was undertaken with the intention of leading to future “mainstream” implementation as a Hybrid 3 study (Fig. 2).

## Results

### Participant demographics

From the 61 operational staff identified across all sites, 32 responded to the invitation (52%) and all were interviewed. Participants came from four Australian states (participated/invited): New South Wales (7/18); Queensland (4/6); South Australia (5/8); Victoria (16/29). The disparity in location representation reflects the proportion of patients recruited to the project from each state. Participants varied by professional role (participated/invited): clinical geneticists (10/24); clinical genetics trainee (4/5); genetic counselors (8/11), intensivists (5/7); laboratory scientists (3/11); project officers (2/3). Reporting by professional role does not include the project officers or laboratory scientists, owing to small sample sizes. See Supplementary Table 1 for a further breakdown of participants' demographics.

### Findings by CFIR domains and constructs

All CFIR domains were reported in the participant interviews, though the Outer Setting was least well represented. The most commonly described CFIR domains across all interviews, indicated by numbers of interviewees or proportion of transcripts dedicated to discussion, were the Inner Setting and Intervention Characteristics. Of particular interest, and providing more nuanced insights, are the constructs that regularly arose across all interviews, regardless of professional role, implementation phase, or location. These CFIR constructs included: “networks and communication”, “implementation leaders”, “culture”, “relative advantage”, and “available resources’.

#### Networks and communication

Overwhelmingly the most commonly reported construct centered on networking and developing relationships. Some focused on communication, e.g., “I did think that the kind of email chain [communication as each stage of the diagnostic process was completed] that happened, that was excellent.” [PO1]. Participants also described the development of relationships, e.g., “I think it has really helped us establish a really good relationship with intensivists where we may not have had as close a working relationship with them in the past” [GC7]. However, there were areas that could be improved, “What would help to have a better relationship would be something like weekly genetic rounds in PICU… At the moment it is still there but ad hoc, and it depends a little bit on that level of suspicion from within ICU team, or the genetics team, as to when a geneticist gets involved.” [I4].

#### Implementation leaders

The agility and success of the program were attributed in part to the program leads, e.g., “I think this is a standout program in a nutshell. It was really the adaptability and the preparedness, whether that’s because of the acuteness of that program, or the leadership—those were the two things which I thought really contributed to this program to be a standout success” [L3]. The extent of the whole multidisciplinary involvement was recognized, e.g., “I know it’s taken a lot of work for lots of other people to get it running that way but it’s been a huge undertaking for the lab people as well as the clinical people and I think the whole process of the Australia-wide leadership team, that’s been incredibly good. They’ve all been really engaged, and it’s moved quickly so I’ve been quite impressed with it.” [CG8].

#### Culture

The different cultures within genetic services and intensive care settings were apparent, e.g., from the genetic services: “They’re [intensivists] not as onboard and our [genetic] counselor would say…‘I really need a sample from this child now,’ and the child would have an indwelling collection line… and the nurse would say, ‘I’m not going to do … I need a doctor’s order for that and I can’t get the doctor because the doctor’s busy.’ So, they might have to literally wait hours to get a sample that could actually take two minutes.” [CG10], and from NICU/PICU: “I still have a bit of a feeling that geneticists come very briefly as guests and do their thing and go again. I don’t think they are as much part of our clinical team as I, personally, think they should be.” [I2]. Trust also featured within the “culture” construct, e.g., “They were clearly busting their guts, to get the time frame. You know, you could see [Program Lead] was getting e-mails at 11pm on a Friday and you know 2am on a Saturday. And to be able to quote that back to the family was tremendously helpful. And not only that but to then trust that that would actually be the case.” [GC7].

#### Relative advantage

Participants noted the benefit of accessing ultra-rapid testing for acutely ill children in comparison with traditional diagnostic approaches that typically yield results over a period of months or years, e.g., “I can’t see why it wouldn’t be useful. I can’t think of a situation where you wouldn’t want to know that [result] quickly.” [I3], and in particular for decisions about care and for the family “It’s just really nice to have this fast test where you can give a result to people quickly and to be able to make management decisions from that, or at least give a family a diagnosis or a reason why this has happened.” [CGT3].

#### Available resources

Occasionally discussion around resources focused on the implications for the financial budget, e.g., “We’re looking forward to more rapid answers and without having to impact on our clinical budget.” [CG10], although more commonly centered on the staff resources, e.g., “the time frames were quite tight, so the turn around times require the pathologists just obviously to be ready and have the capacity.” [PO2]. There was also an awareness of the potential for ultra-rapid genomics to alter resource allocation in the healthcare system on a broader scale, e.g., “Obviously, there’s a massive financial side to it, you know. When you stay, when you keep someone alive, for example, for, you know, four to six weeks waiting for an answer, I’ve been in that situation before, so my expectations were that a three to four day turnaround was going to make really significant management changes. There was certainly going to be some babies [where it would] influence withdrawal of care decisions.” [CG5].

The CFIR constructs of “networks and communication”, “implementation leaders”, “culture” “relative advantage”, and “available resources“ were found across the interviews. However, findings by CFIR constructs varied by professional role and implementation phase.

### Differences by professional role (see Fig. 3 for exemplar quotes)

In addition to the CFIR constructs listed above, clinical geneticists raised the importance of “design quality & packaging” to ensure rapid genomic testing was focused on the family, e.g., the process of informed consent being appropriately tailored to the acute care environment. Although the importance of “networks and communication” was a theme across professional roles, it was an overriding area of discussion for genetic counselors. This professional group stressed the value of developing local and national networks, in person and virtually, for relationship building as key for the long-term compatibility of the program, and also for knowledge transfer. The genetic counselors were highly involved with many of the logistical, patient-facing challenges, such as getting samples to the laboratories and so, perhaps unsurprisingly, also reported “executing” as a strong theme. In addition to some of the constructs common to all professional roles, the intensivists reported “accessing knowledge”, in particular from the clinical geneticists and clinical genetics trainees, to facilitate decision making.

### Differences by implementation phase (see Fig. 4 for exemplar quotes)

The reported CFIR constructs varied in prominence across implementation phases. Pre-implementation the focus was on the “relative advantage” of using ultra-rapid genomics in providing a timely diagnosis for families with critically ill children. Concerns at this early stage primarily centered around “design quality & packaging” with some apprehension about how compatible the program was with regular clinical activity, in particular how to support staff delivering the ultra-rapid service. Post-implementation “networks and communication” were noted to have played a key role in engagement. The perceived fit of ultra-rapid genomics was, at times, challenged by the “design quality & packaging”, especially in relation to ensuring the service was designed around the families, who are often in a highly distressed state due to the critical nature of the illness in their child^24^. The success of the project was also determined by “other personal attributes”, including self-motivation and enthusiasm for the provision of ultra-rapid genomics for this patient group.

Looking to the future and considering the sustainability of the program, participants again noted the importance of “networks and communication”, centering on the building of relationships and developing networks. Open and transparent communication enabled participants to feel engaged with the project. Having the “available resources” of time and appropriate skills was seen as essential to longevity, in particular, those required for counseling,

## Discussion

Using the CFIR, we identified many themes at the forefront of professionals’ perceptions of how ultra-rapid genomic testing fits into their practice, regardless of professional role or implementation phase. In common with other studies^16,25^, the construct “networks and communication” was a recurrent theme. However, there was an evolution from our initial Hybrid 1 effectiveness-implementation study (Fig. 2)^11^, where the focus was on the process communications required, for example, how to manage samples through a new pathway. The construct has now progressed to a broader consideration of relationships and how to spread knowledge and experience between professional groups and between geographically dispersed sites, setting up a feedback loop to understand outcomes, which in turn can influence adoption and fidelity.

There was a strong cultural theme, demanding a broadening of the standard CFIR definition of the “culture” construct to ensure these findings were fully represented (Supplementary Table 2). The Australian Genomics Acute Care program was dependent on bringing together multiple professional groups from disparate organizations in a time-pressured and emotionally charged environment. The significance of understanding each other’s evolving roles and developing trust was evident. Building, and more importantly, maintaining trust^26^ is crucial to scaling up a program and is dependent on delivering what is promised: a shared understanding and integrity^27^. These three features were demonstrated by consistently delivering the target turnaround time of under 5 days, and through transparency by providing both ‘live’ feedback as cases progressed through the steps of the testing pathway, and on regular feedback to the leadership group.

The role of leadership was singled out for attention, recognizing the importance of leadership flexibility and promoting inclusivity. Leadership is a relatively new discussion point in the implementation of clinical genomics^28^, and the traits identified here are associated with proactively delivering innovative and dynamic projects. Taking a more controlled “top-down” leadership approach might successfully deliver a project that demands high levels of accuracy at multiple levels under tight time constraints, however, this type of rigid hierarchical strategy discourages taking responsibility locally and encourages disparate workarounds. A distributed leadership approach with identified and accessible fora for advancing team knowledge-sharing helped promote consistency of priorities and shared values.

**Fig. 3:**
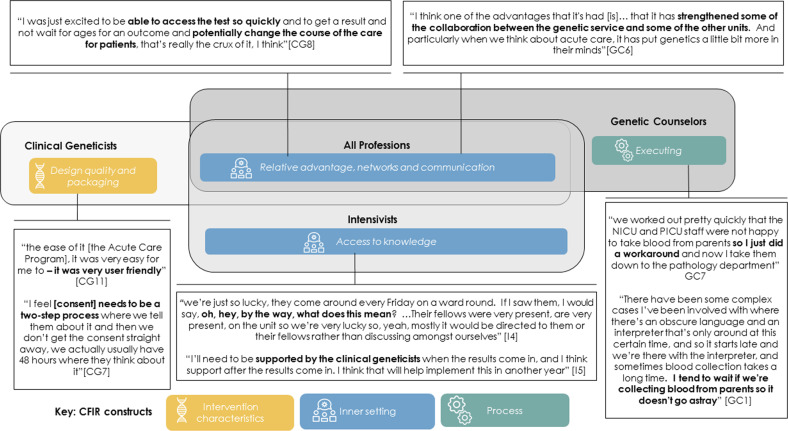
Differences by profession for findings by Consolidated Framework for Implementation Research (CFIR) domains and constructs, with exemplar quotes. Three CFIR domains (with their associated constructs) are reported: intervention characteristics (design quality and packaging), inner setting (access to knowledge, relative advantage, networks and communications) and process (executing).

**Fig. 4:**
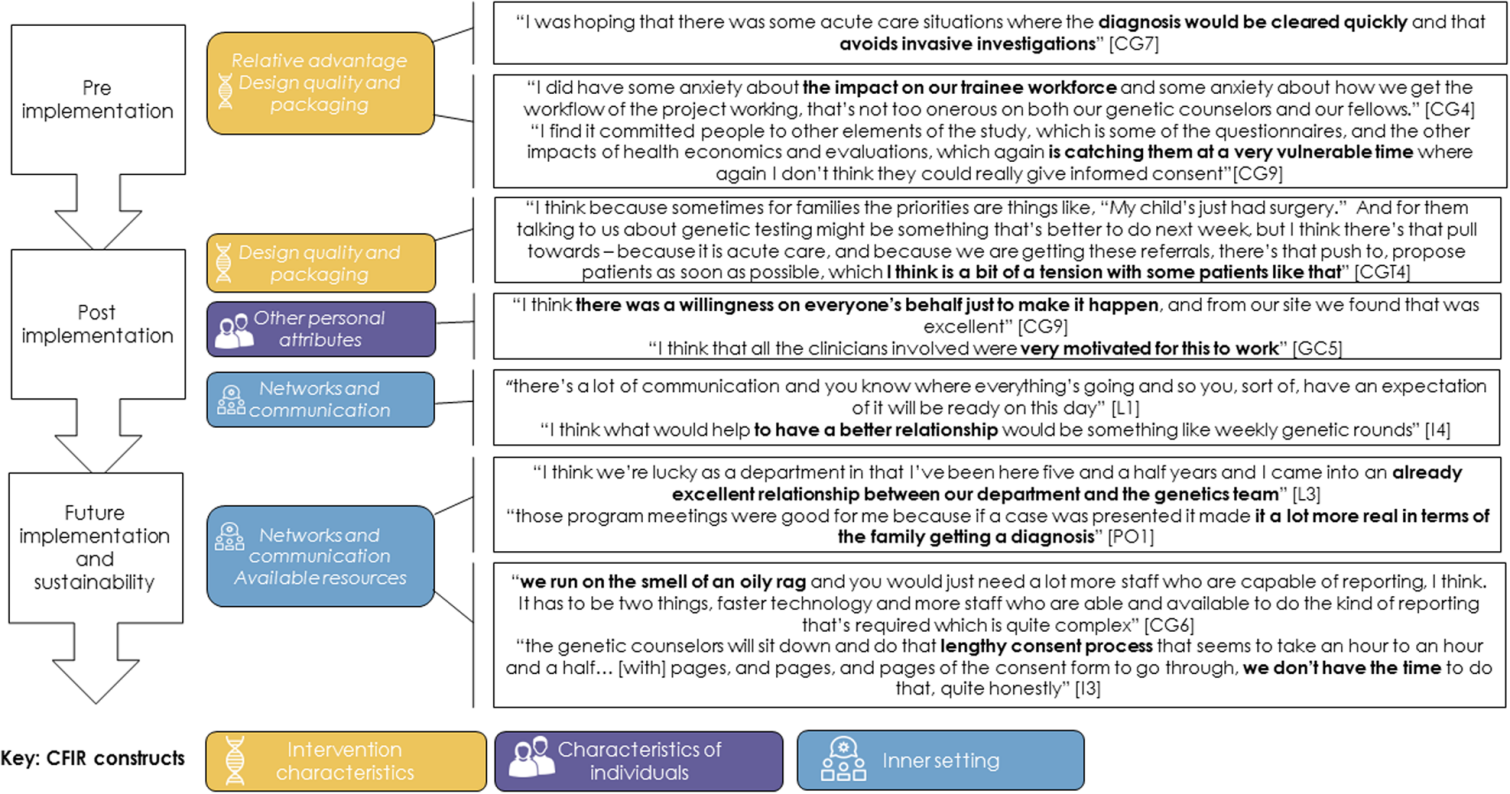
Differences by implementation phase for findings by Consolidated Framework for Implementation Research (CFIR) domains and constructs, with exemplar quotes. Three CFIR domains (with their associated constructs) are reported: intervention characteristics (relative advantage, design quality and packaging), inner setting (available resources, networks and communications) and characteristics of individuals (other personal attributes).

Some CFIR themes varied by professional role or implementation phase, or both. Future implementation strategies will benefit from modifications to professional roles and implementation stages, in order to optimize professional engagement and efficiencies (Figs 3 and 4). Clinical geneticists were specifically focused on Intervention Characteristics and the “design quality and packaging” of ultra-rapid genomic testing. Future implementation strategies should ensure a focus on family needs and preferences while maintaining clinical efficacy^29,30^. Streamlined processes for data capture and integration could be supported by regular monitoring of diagnostic and clinical outcomes and timely feedback to clinicians. Genetic counselors reported the Process construct of “executing”, which will likely be of concern in new locations where rapid genomic testing is not yet embedded into clinical care. Teams at sites implementing ultra-rapid genomic testing for the first time can be supported by those at established sites, though their own “executing” challenges will be nuanced by their local environment. Intensivists highlighted the importance of the Inner Setting construct of “access to knowledge” and, in particular, the interaction with clinical geneticists. Over time, intensivists may become more confident and require less support, particularly as genomic testing becomes increasingly part of mainstream pediatric care, although this transition was not indicated in the interviews. Some intensivists may require genomics education, and the use of peer influence or opinion leaders may be appropriate.

Across the recurrent themes through the implementation phases, an interesting pattern emerged (Fig. 4). At the pre-implementation stage, attention was firmly focused on Intervention Characteristics such as the “relative advantage” of using ultra-rapid genomic testing instead of traditional diagnostic approaches. Post-implementation, a more complex picture emerged, with a mix of CFIR domains and constructs reported. The importance of “personal attributes” such as motivation, commitment, and willingness was described, which may not be sustainable longer term. When considering sustainable models of care in the future, a consensus was again evident and was highly dependent on Inner Setting constructs such as building “networks and communication”. This finding demonstrates that priorities shift over the lifetime of the project and priorities that are important early on, when creating engagement, are different to priorities for sustaining a project longer term. Implementation strategies will need to be adaptive^31^, dependent on the implementation phase, and implementation leaders will need to be aware of this shift in focus in order to maximize project engagement.

**Fig. 5:**
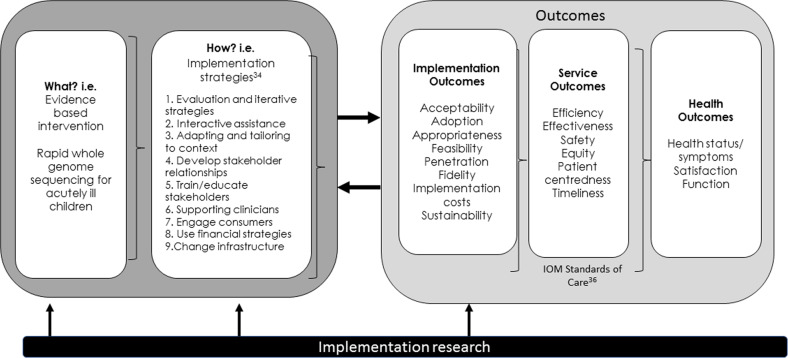
Identifying implementation strategies and outcomes using the Conceptual Model of Implementation Research^32,41^. This step by step model first identifies ‘what’ is being implemented (here, rapid whole genome sequencing for acutely ill children). The ‘how’ then lists potential implementation strategies that could support implementation. The ‘what’ and the ‘how’ lead to three programme outcomes – Implementation, Service and Health.

Before determining appropriate implementation strategies, it is essential to consider the intended outcomes. Used alongside the effectiveness-implementation hybrid model^11^, the Conceptual Model of Implementation Research^32^ (Fig. 5) allows a clear articulation of implementation research amidst a clinical program. Having identified the evidence based practice to implement, the next step is to identify implementation strategies, guided by practice taxonomies^33,34^ and findings from previous studies, to address the intended outcomes, i.e., the impact or effect (rather than the product or output) delivered by a service, treatment or practice. Outcomes include: (1) implementation, i.e., the impact of implementation strategies; (2) service, i.e., the effect on the healthcare system; (3) health, i.e., the effect on clinical or quality of care measures.

From this study, we have identified a range of implementation strategies, for example, building relationships and maintaining open and transparent communications (“networks and communication”) and revising professional roles to enable staff to take up different responsibilities (“available resources“) and identified relevant outcomes. Table 1 highlights how priorities shift by professional role and phase of implementation and identifies a proposed theory-informed implementation strategy^33^ with an example of how this may look, complete with the proposed outcome. For example, the clinical geneticists were focused on the intervention characteristics of the design quality and packaging of ultra-rapid genomic testing to ensure it is patient-centered. An implementation strategy of regular audits with feedback would provide transparent feedback on clinical outcomes and provide information on the implementation outcome of effectiveness. In the next iteration of the project, we will compile these approaches into categories (e.g., planning, education, financing, restructuring, quality management, and attention to policy contexts)^33,35^ to provide a comprehensive range of strategies on which to draw. Stakeholders can then be invited to rate the implementation and feasibility of the potential implementation strategies using concept mapping techniques^36^.

**Table 1:** Examples of proposed implementation strategies and intended outcomes by professional role and implementation phase.

Role/phase	Priority by Consolidated Framework for Implementation Research (CFIR) code—domain/construct	For example,	Proposed implementation strategy for future implementation taken from the Expert Recommendations for Implementing Change (ERIC)^33^ taxonomy	For example	Proposed outcome I = implementation H = Health S = Service
All	Inner setting/networks and communication	Building relationships	Develop stakeholder relationships	Regular communication and feedback with the team. Build external relationships	I: Penetration I: Sustainability
Genetic counselors	Process/executing	Ensuring local processes meet the ultra-rapid time constraints	Support clinicians/change infrastructure	Experienced genetic counselors mentor those new to ultra-rapid genomics	I: Fidelity
Clinical geneticists	Intervention characteristics/design quality and packaging	Patient centred program design	Evaluation and iterative strategies (audit and feedback)	Transparent and regular feedback on clinical outcomes	S: Effectiveness
Intensivists	Inner setting/access to knowledge	Access to genomic knowledge	Revise professional roles	Upskill compatible non-genetic medical specialists e.g., endocrinologists/neurologists	I: Feasibility
Pre-implementation	Intervention characteristics/relative advantage	Getting rapid answers for families	Engage consumers	Development of a family report	I: Acceptability
Mid-implementation	Characteristics of individuals/other personal attributes	Motivation	Develop stakeholder relationships	Support local champions	I: Sustainability
Sustainability	Inner setting/available resources	Access to counseling	Adapting and tailoring to context	Use of telehealth	I: Penetration

Future scaling up of the Australian Acute Care Genomics program will progress into a Hybrid 3 design (Fig. 2) as clinical effectiveness of ultra-rapid genomic testing becomes established^1–6,19^. This provides an opportunity to employ and test relevant implementation strategies, drawing on quantitative and qualitative data, while ensuring ongoing clinical efficacy.

This study has limitations. Just over half of those invited to participate took part, and we had a clear representation across most professional roles, although there was limited input from the laboratory scientists. All sites participating in the Australian Genomics Acute Care program did so on a voluntary basis, and expressed high levels of implementation readiness prior to study commencement^37^. Sites that declined participation, and those that recruited relatively few patients would provide an interesting group for further study. Qualitative research typically involves relatively lower levels of participants than quantitative research, as it does in this case, but allows more in-depth examination of issues. Worldwide, approaches to implementation vary. This study was undertaken in one country, from a public healthcare system perspective, and so caution may be required when interpreting or applying these findings elsewhere. The CFIR is challenging to apply consistently in practice and is time intensive, potentially delaying the identification of findings and therefore the application of relevant intervention strategies^38^. Although our interview schedule was intentionally broad and centered on participant perceptions to promote open discussion, there were limited findings related to the CFIR domain Outer setting. This absence does not mean the economic, political, and social context is not relevant, as it may not typically be the immediate focus of participants such as ours. A focus on understanding this area may be of benefit in future studies. In addition, participants in this study were patient-focused and did not report costs as a barrier as costs are covered by the research study. The significance of resource consumption merits further detailed health economic analysis and additional investigation with both policymakers and organizational leads.

Combining implementation science principles, genomic medicine ideals and learning healthcare system concepts is helping shape new models of care by which we can apply the findings from biomedical research and can contribute value to the rapidly evolving healthcare environment^9^. Investigating the design and scale-up of ultra-rapid genomic testing in critical care pediatrics across multiple sites using implementation science principles and theory reveals many shared priorities of stakeholders, such as “networks and communication”, where common approaches can be employed. Nevertheless, there are differences specific to professional roles and to implementation phases which will benefit from tailoring of implementation strategies to optimize the potential of ultra-rapid genomic testing. This study of professional perspectives forms an important part of the evaluation of rapid genomic testing in pediatrics and is complemented by parental experiences^29^ and diagnostic outcomes^19^. The findings from these three areas will support the further development of robust, context-specific implementation strategies that can be applied and tested as we move towards sustainable healthcare system implementation.

## Methods

### Participants and recruitment

One year after the Australian Genomics Acute Care program commenced, the implementation leaders at each site identified all operational staff delivering the program, including clinical geneticists, clinical genetics trainees, genetic counselors, intensivists, project officers, and laboratory scientists. These staffs were invited to participate in an interview via email (SB), with up to two follow-up invitation e-mails. Owing to slow uptake by laboratory scientists, the Acute Care program lead (ZS) sent one additional email to this group encouraging them to contact the lead researcher directly. Participation was voluntary and only the lead researcher (SB) was aware of participant identities. Ethical oversight was provided by the University of Melbourne, Department of Pediatrics, Research Ethics Committee (HREC Number: 1646785).

### Data collection tools and procedure

A cross-sectional, exploratory qualitative approach was used^39^. We designed an open semi-structured interview schedule to investigate perceptions by promoting discussion along with implementation phases. Questions centered on what participants expected before taking part in program delivery, how they thought the Acute Care program would fit into their current practice, reflections following implementation, and their thoughts on sustainability for the future. For consistency, one researcher (SB) undertook all interviews, from April to June 2019, either face-to-face or via telephone, dependent on participant location and preference. The interviews averaged 30 minutes duration, were audio-recorded with participants’ informed verbal consent (we did not take written consent), fully transcribed and managed in NVivo 12^40^. Transcripts were de-identified, numbered and given a unique classification code by professional role (clinical geneticists [CG], clinical genetics trainees [CGT], genetic counselors [GC], intensivists [I], project officers [PO], and laboratory scientists [L]).

### Data analysis

Interview data were coded in two steps, to overcome established challenges in consistently and efficiently applying the domains and constructs of the CFIR^38^. Step 1: applying the five overarching CFIR domains, and Step 2: interrogation of each CFIR domain by the underlying constructs. Before coding, the CFIR domains and constructs were mapped to the Australian Genomics Acute Care program (Supplementary Table 2). During Step 1, two experienced qualitative researchers (SB and HB) undertook the mainstay of the coding and met regularly throughout data analysis to ensure internal consistency. Initially, ten transcripts were coded independently for the CFIR domains, and findings were compared to discuss and resolve any discrepancies. SB then completed the remainder of Step 1. During Step 2, one CFIR domain, Intervention Characteristics, was coded independently (SB and HB) to identify constructs, with any differences discussed. Coding of the remaining constructs was then completed by SB, in ongoing consultation with HB. For any challenging or unresolved areas of coding, a third investigator (BM) was available to achieve a resolution. Implications from the commonly reported CFIR constructs were considered, including any differences by professional role and implementation phase, to identify how these findings can help inform future iterative development of the Australian Genomics Acute Care program.

### Reporting summary

Further information on research design is available in the Nature Research Reporting Summary linked to this article.

## Supplementary information

Supplementary Information

Reporting Summary

## Data Availability

Further data that support the findings of this study are available in Supplementary Table 3. Full data are not publicly available as participants consented to share relevant quotes and not full interview transcripts with third parties.

## Code availability

Code availability is not applicable to this study
