# Supplementary material for: Learning from scaling up ultra-rapid genomic testing for critically ill children to a national level
Source: NPJ Genom Med. 2021 Jan 28;6:5. doi: 10.1038/s41525-020-00168-3 (PMC7843635; doi:10.1038/s41525-020-00168-3)
Supplement: Supplementary file 1 — Supplementary Information [file 41525_2020_168_MOESM1_ESM.pdf]

Supplementary Table 1: Characteristics by state. * Population data taken from the Australian Bureau of Statistics as of 2019 https://www.abs.gov.au/AUSSTATS/abs@.nsf/mf/3101.0

**Location**	**NSW**	**QLD**	**SA**	**VIC**
Population (in '000)*	8129.0	5130.0	1759.2	6651.1
Interviewees: participated/total invited	7/18	5/6	4/8	16/29
Genetic staff (clinical geneticists, clinical genetics trainees and genetic counsellors). Participated/total invited	5/12	2/4	4/6	11/18
Nongenetic staff (intensivist, laboratory scientists and project officers). Participated/total invited	2/6	2/2	1/2	5/11
No. patients referred for testing (total N=108)	29	8	8	63
Number of participating hospitals, (number of NICU and PICU beds)	6 (234)	2 (105)	1 (45)	3 (205)
Number of participating laboratories	1	0	0	1
Time from hospital admission to consent in days, mean (95% confidence interval)	12.1 (8.45-17.8)	18.1 (9.38-33.7)	17.8 (4.5-65.1)	11.6 (8.7-15.9)

Supplementary Table 2: Consolidated Framework for Implementation Research (CFIR) coding applied to the acute care context

Key: UR - ultra-rapid

CFIR constructs by domain		Short description	Description in acute care context
**I. INTERVENTION CHARACTERISTICS**			
A	Intervention source	Perception of key stakeholders about whether the intervention is externally or internally developed.	Perceptions of key stakeholders about whether the use of UR genomics in acute care is externally or internally developed.
B	Evidence strength & quality	Stakeholders' perceptions of the quality and validity of evidence supporting the belief that the intervention will have desired outcomes.	Stakeholders' perceptions of the quality and validity of evidence supporting the belief that the use of UR genomics in acute care will have desired outcomes
C	Relative advantage	Stakeholders' perception of the advantage of implementing the intervention versus an alternative solution.	Stakeholders' perception of the advantage of implementing UR genomics in acute care versus an alternative solution.
D	Adaptability	The degree to which an intervention can be adapted, tailored, refined, or reinvented to meet local needs.	The degree to which UR genomics in acute care can be adapted, tailored, refined, or reinvented to meet clinical, lab, operational or patient needs.
E	Trialability	The ability to test the intervention on a small scale in the organization, and to be able to reverse course (undo implementation) if warranted.	The ability to try out UR genomics in acute care and to be able to reverse course (undo implementation) if warranted.
F	Complexity	Perceived difficulty of implementation, reflected by duration, scope, radicalness, disruptiveness, centrality, and intricacy and number of steps required to implement.	Perceived difficulty of implementation UR genomics in acute care, including duration, scope, radicalness, disruptiveness, centrality, and intricacy and number of steps required.
G	Design quality & packaging	Perceived excellence in how the intervention is bundled, presented, and assembled.	Perception of how UR genomics in acute care is presented and assembled.
H	Cost	Costs of the intervention and costs associated with implementing the intervention including investment, supply, and opportunity costs.	Costs of UR genomics in acute care, plus associated costs e.g. investment, supply, and opportunity costs.
**II. OUTER SETTING**			
A	Patient needs & resources	The extent to which patient needs, as well as barriers and facilitators to meet those needs, are accurately known and prioritized by the organization.	The extent to which patient and family needs (including barriers and facilitators to meet those needs), are accurately known and prioritised by the organizations involved and the acute care team.
B	Cosmopolitanism	The degree to which an organization is networked with other external organizations.	The degree to which the UR program organisations and acute care team are networked with other external organizations.

C	Peer pressure	Mimetic or competitive pressure to implement an intervention; typically because most or other key peer or competing organizations have already implemented or are in a bid for a competitive edge.	Mimetic or competitive pressure to implement UR genomics in acute care; *typically, but not always,* because most or other key peer or competing organizations have already implemented or are in a bid for a competitive edge.
D	External policy & incentives	A broad construct that includes external strategies to spread interventions, including policy and regulations (governmental or other central entity), external mandates, recommendations and guidelines, pay-for-performance, collaboratives, and public or benchmark reporting.	A broad construct that includes strategies external to the program, to spread interventions, including policy and regulations (governmental or other central entity), external mandates, recommendations and guidelines, pay-for-performance, collaboratives, and public or benchmark reporting.
**III. INNER SETTING**			
A	Structural characteristics	The social architecture, age, maturity, and size of an organization.	The social architecture, age, maturity, and size of the organization and the acute care team.
B	Networks & communications	The nature and quality of webs of social networks and the nature and quality of formal and informal communications within an organization.	Nature and quality of i) social networks and ii) formal and informal communications within either the home organisation or the acute care team
C	Culture	Norms, values, and basic assumptions of a given organization.	Norms, values including trust, and basic assumptions of either the home organisation or the acute care team.
D	Implementation climate	The absorptive capacity for change, shared receptivity of involved individuals to an intervention, and the extent to which use of that intervention will be rewarded, supported, and expected within their organization.	The absorptive capacity for change, shared receptivity of involved individuals to UR genomics in acute care, and the extent to which use of UR genomics in acute care will be rewarded, supported, and/or expected within their organization.
1	*Tension for change*	The degree to which stakeholders perceive the current situation as intolerable or needing change.	The degree to which stakeholders perceive the lack of UR genomics in acute care as intolerable or needing change.
2	*Compatibility*	The degree of tangible fit between meaning and values attached to the intervention by involved individuals, how those align with individuals' own norms, values, and perceived risks and needs, and how the intervention fits with existing workflows and systems.	Values attached to UR genomics in acute care aligned with personal norms and values, perceived risks and needs, and how UR genomics in acute care fits with current ways of working and systems
3	*Relative priority*	Individuals' shared perception of the importance of the implementation within the organization.	Individuals' shared perception of the importance of the implementation of UR genomics in acute care in the organization.
4	*Organizational incentives & rewards*	Extrinsic incentives such as goal-sharing awards, performance reviews, promotions, and raises in salary, and less tangible incentives such as increased stature or respect.	Incentives external to the program e.g. awards, performance reviews and less tangible incentives e.g. respect,

5	*Goals and feedback*	The degree to which goals are clearly communicated, acted upon, and fed back to staff, and alignment of that feedback with goals.	The degree to which UR genomic goals are clearly communicated, acted upon, and fed back to staff, and alignment of that feedback with goals.
6	*Learning climate*	A climate in which: a) leaders express their own fallibility and need for team members' assistance and input; b) team members feel that they are essential, valued, and knowledgeable partners in the change process; c) individuals feel psychologically safe to try new methods; and d) there is sufficient time and space for reflective thinking and evaluation.	The climate around UR genomics that allows: a) leaders express their own fallibility and need for team members' assistance and input; b) team members feel that they are essential, valued, and knowledgeable partners in the change process; c) individuals feel psychologically safe to try new methods; and d) there is sufficient time and space for reflective thinking and evaluation.
E	Readiness for implementation	Tangible and immediate indicators of organizational commitment to its decision to implement an intervention.	Overt indication that an organization is committed to implement UR genomics in acute care
1	*Leadership engagement*	Commitment, involvement, and accountability of leaders and managers with the implementation.	Commitment, involvement, and accountability of leaders and managers with the implementation of UR genomics in acute care
2	*Available resources*	The level of resources dedicated for implementation and on-going operations, including money, training, education, physical space, and time.	Resources dedicated for implementation of UR genomics and on-going operations, including money, training, education, physical space, and time.
3	*Access to knowledge & information*	Ease of access to digestible information and knowledge about the intervention and how to incorporate it into work tasks.	Ease of access to digestible information and knowledge about UR genomics in acute care and how to incorporate it into work tasks.
**IV. CHARACTERISTICS OF INDIVIDUALS**			
A	Knowledge & beliefs about the intervention	Individuals' attitudes toward and value placed on the intervention as well as familiarity with facts, truths, and principles related to the intervention.	Individuals' attitudes toward and value placed on the use of UR genomics in acute care as well as familiarity with facts, truths, and principles related to the intervention.
B	Self-efficacy	Individual belief in their own capabilities to execute courses of action to achieve implementation goals.	Individual belief in their own capabilities to deliver UR genomics in acute care
C	Individual stage of change	Characterization of the phase an individual is in, as he or she progresses toward skilled, enthusiastic, and sustained use of the intervention.	The phase of an individual as they progress to becoming skilled and enthusiastic about sustained use of UR genomics in acute care
D	Individual identification with organization	A broad construct related to how individuals perceive the organization, and their relationship and degree of commitment with that organization.	How the individual perceives their organisation or the acute care team, and the extent of the commitment with their organisation or the acute care team

E	Other personal attributes	A broad construct to include other personal traits such as tolerance of ambiguity, intellectual ability, motivation, values, competence, capacity, and learning style.	Personal traits e.g. tolerance of ambiguity, intellectual ability, motivation, values, competence, capacity, and learning style.
**V. PROCESS**			
A	Planning	The degree to which a scheme or method of behavior and tasks for implementing an intervention are developed in advance, and the quality of those schemes or methods.	The degree of planning that has taken place before the implementation of UR genomics in acute and the quality of the planning
B	Engaging	Attracting and involving appropriate individuals in the implementation and use of the intervention through a combined strategy of social marketing, education, role modeling, training, and other similar activities.	The process of engaging others in the implementation of UR genomics in acute care, e.g. social marketing, education, role modeling etc
1	*Opinion leaders*	Individuals in an organization who have formal or informal influence on the attitudes and beliefs of their colleagues with respect to implementing the intervention.	People in the organization or acute care team who have influence over their colleagues relating to the implementation of UR genomics in acute care
2	*Formally appointed internal implementation leaders*	Individuals from within the organization who have been formally appointed with responsibility for implementing an intervention as coordinator, project manager, team leader, or other similar role.	Implementation leadership including anyone involved with the implementation of the program. Including roles such as e.g. coordinator, project manager, team leader
3	*Champions*	Individuals who dedicate themselves to supporting, marketing, and ‘driving through’ an intervention, overcoming indifference or resistance that the intervention may provoke in an organization.	Individuals who dedicate themselves to supporting, marketing, and ‘driving through’ UR genomics in acute care, overcoming indifference or resistance that the intervention may provoke in an organization.
4	*External change agents*	Individuals who are affiliated with an outside entity who formally influence or facilitate intervention decisions in a desirable direction.	People from outside the organization or acute care team who formally and positively influence or enable implementation of UR genomics in acute care
C	Executing	Carrying out or accomplishing the implementation according to plan.	The process of implementing UR genomics in line with the plan
D	Reflecting & evaluating	Quantitative and qualitative feedback about the progress and quality of implementation accompanied with regular personal and team debriefing about progress and experience.	Feedback about how implementation of UR genomics in acute care is progressing including personal and team perspectives

Supplementary Table 3: Exemplar quotes by assigned Consolidated Framework for Implementation Research (CFIR) domain

Note: Definition in context taken from Supplementary table 3

Overall

CFIR construct: networks and communication	*Comments*
**Definition in context:** Nature and quality of i) social networks and ii) formal and informal communications within either the home organisation or the acute care team	
We also have the option to join in those, the variant prioritisation discussions. Although we don't have to, but we're invited, so if we want to. GC1	Communication processes (also links to culture)
I was able to work out right the results are most likely going to be ready Monday morning, let me factor that into my day on Monday that we're going to have to go a deliver the results at some stage on Monday. It made me feel included. A lot of the time as a genetics trainee we're not necessarily deliberately excluded, but we're not included as much as what other clinical geneticists who are specialists are included, and I think having the email trails include us when we had a patient involved was good CGF2	Inclusion in the intake and variant prioritisation meetings
We have got a genetic counsellor thread and then there's the actual Acute Care thread which includes the lab staff and all of that. So what happens is at each stage we say, "We are getting consent at this time," then we know that's when we are going to be going down. And then, when we have done that, we can say actually we can expect samples at this time or with da da da da da. So my feeling has been that that has been a very good tool to educate. GC2	Communication (ties to building trust)
The [monthly project] teleconference and look at the filtering and interpretation of the variants, so we don't really get much of a chance to do that in [state name], so being part of that was really good and I feel like that's something we should do more of here and we're trying to do it just in general, day-to-day things with our own lab. CGT3	Communication process (leads to building confidence in capabilities)
having a genetic counsellor that is going up to the NICU and PICU wards quite consistently is something new for them and getting used to how a genetic counsellor works and what we actually do so I think that's been really, really good, just raising awareness of genetic counselling as a whole. GC7	Communication building relationships

**CFIR construct: implementation leaders**	
**Definition in context:** Implementation leadership including anyone involved with the implementation of the program. Including roles such as e.g. coordinator, project manager, team leader	
I'm sure [program lead] would have wanted me to be state lead, or did want me to be state lead. But I actually just couldn't commit to having more and more and more things on my plate, and for political reasons, I thought it would be good to give [current state lead] a kind of a lead in something. CG10	Consideration given to appointing leads
[state lead name] is now the biggest state lead for the project, so he was also instrumental in helping me with pathology, so the meetings set up with the key pathology people and [state lead name] which helped a lot to have that kind of clinical link.PO1	Role of lead (links to networking too)
I think the consultants are all fairly consistent, and I think [program lead] helps us with that [talking with NICU/PICU]. She's always very open to considering our patients and discussing those if there's any uncertainty from the consultants involved. CGT4	Role of lead
the head of department is very pro genomic testing at our place, so [name of head of department] was head of the department at the time, very, very pro genomic testing, involved in a lot of cerebral palsy genomic research, is very, very, very proactive in understanding what's going on with her patients. So for her it was a – and – for her it was a real bonus, and she - that's the attitude in that department that knowledge is important, this knowledge is important CG10	Influence of department leads on take up (ties to knowledge and beliefs about the intervention)
the general sense is that I think that [Organisation name] has been fantastic to kick start all this conversation, and it's got a whole lot of people taking notice, and it's moving in absolutely in the right direction with the right leadership CG9	Importance of leadership on setting direction
The human side went well, it's really [program lead] and I have had some experience with [program lead] in other gene or genomics efforts mostly on a family by family basis, she referred a few families to us, we help, she's super organised, she's reasonably quiet but a very determined achiever so I think the leadership here on her behalf was the key to success in my opinion L3	Approach to leadership (ties to networks and building relationships)
I think it has all worked incredibly well. There was nothing negative from my perspective about the flagship and how it's been conducted. And I guess a lot of that was [program lead], played a major, major role, I think, in bringing everyone together. But it seemed that all of the parts of the flagship were equally committed, and I think that's why it's worked so well. CG8	Role of lead in engaging the team

How do we organise the parents' blood because on the ward they don't always like doing it, or they don't like – it was a bit of work to get the teams on the ward to see that it was important. Well so we've got [program lead] haven't we? GC1	Supportive leadership role (ties to culture)
CFIR construct: culture	
**Definition in context:** Norms, values including trust, and basic assumptions of either the home organisation or the acute care team.	
as soon as you bring a few research minded people suddenly you start seeing something which the clinical and the diagnostic force simply doesn't see. L3	Building team norms
basically every single person throughout the lab has stood up and said, "We're willing to do extraordinary things that go above and beyond the call of duty for what our job description says," and everyone just bought into it, so that's great we've got a fantastic team, but it does still sort of leave this residual question of, "Well, is that reproducible elsewhere? Can you just roll that out in a routine healthcare scenario?" where staff are just working nine to five and, you know, you just can't just assume that that's scalable. L2	Values within the team
I thought the multidisciplinary team approach worked really, really well, we seemed to have a lot of – all the NICU staff were on board with the project, the communication was really good, the response time from the acute care team was phenomenal though I think that the processes that had been put in place beforehand in terms of communication and laboratory requirements and things like that was fantastic. GC5	Team culture established within genetics dept and into NICU
I think the most notable thing in terms of our success here has been how tight knit the team that's done it has been, and so I think that's what I would mostly recommend is that if anyone is setting this up, you need a small focused multidisciplinary team to work on it at a project level - you're not going to be able to easily do this if, you know, you try and do it by just treating it like a normal inter-departmental project that's like when you have a meeting once a month; it just isn't going to work like that. There's too many complexities and there's too much flexibility needed where both sides may have to adjust what they do to come and meet and do things in anyway, so that's the main thing I think is to really take in the very, very flexible approach to it, and the easiest way to do that is with a really focused tight knit group of people. L2	Norms and values
People see a genetic blood and they're so used to that being a not urgent thing that sometimes it could get overlooked for a day or two which of course in an acute care setting is unacceptable - so it was, yeah, politely going back up to the ward and saying, "Oh, did you see that on there?" "Yeah, yeah, we'll get to that." "Oh, did you know that actually things are changing a bit in genetics," and I found that people are very receptive to if you take the time to bring them	Previous norms – genetics not a priority activity. Now changed. (ties to network and communication

on the journey with you as opposed to just saying, "I need this now," if you say why and that's certainly something I learnt while setting up [Organisation name] when talking at the laboratory staff who'd never had genetics need to be a rapid thing and saying to them, "Oh, it's actually because there's a sick baby who this could affect their outcomes," and the lab staff, "Oh, really? Okay, yeah, we will hurry that one along." So bringing people on the journey in a polite way. GC3	and knowledge and beliefs about the intervention)
In the early days I think the pushback [from NICU staff] was around getting blood samples from the parents and things like that. GC2	Early days norms not established
CFIR construct: Relative advantage	
Definition in context: Stakeholders' perception of the advantage of implementing UR genomics in acute care versus an alternative solution.	
I think there's the power of the diagnosis to influence care including withdrawal of care is a really important concept and the power of a negative exome as well. There are situations where with a very volatile child who's unwell, there are various differential diagnoses floating around that sometimes having a negative genomic test result can help care, even though it feels a bit counter-intuitive, 'cause it may be, to the best of our ability rules out certain diagnoses or makes them less likely and it can help the treating clinicians feel more sure that they've thought about and covered off certain diagnostic possibilities.CG4	Perceives rapid genomics is advantageous for their population, even when the news is not positive
At present the real direct impact it has on management is decisions on withdrawing care, which I think is important and the families have appreciated as well, and there maybe even the cost maybe aspect. But I think for the next stage of research it's important to keep in mind that we probably want to go with that further towards personalised medicine that actually allow us personalised treatment decisions that allow patients to survive better. I4	Perception of advantage but feels this could go further
We were able to get results and for some families it was very good to know that and gave them some choices that they would not have had before. So we're not sort of floundering around in the dark guessing at what's wrong with their child, and we can talk to them CG10	Providing answers for families quickly - perceives advantage in relation to standard or no genomic testing
CFIR construct: Available resources	
Definition in context: Resources dedicated for implementation of UR genomics and on-going operations, including money, training, education, physical space, and time.	
I'm the only one that's employed by [Organisation name] and then at the two [two hospital names] the genetic counsellors here will back up if I ask them to so obviously I can't be in two places at once so if needed they'll provide back-up but generally out of the thirty-odd patients or so that have been recruited from [our state] I would have been the majority of them. GC7	Need for genetic counselling resources

I think that's [provision of counseling] been a really crucial part of evolving this test. It didn't just come with it, here's a new, you know, blood or saliva test you can do. It came with all of this associated service and it could easily have not have – I could imagine other, maybe, tight-fisted perhaps services suggesting that you didn't need to have that other holistic aspect of it, but it's critical. I3	Struggle to deliver the service without genetic counselling resource
From a service provision point of view the thing that's concerned me has been the fact that there hasn't been adequate funding for clinical staff to provide this service and although there is obviously an enthusiasm and a commitment to provide it the fellows particularly were in after hours and weekends and things when they weren't paid CG8	Concern if not resourced properly the impact it could have on staff
I think you need a good team of people and that involves a geneticist and a genetic counsellor CG7	Multidisciplinary staffed team required to deliver the service
That's [ultra rapid genomics] very labour intensive and time intensive. CG9	Demands a lot of resources
we were very concerned how that [delivery of the acute care program] would impact on workforce for us, from our point of view and we were going to deliver, within the timeframe of our working week. Particularly because a lot of the geneticists are part-time. So somebody recruits somebody on a Tuesday, they're not necessarily there on a Wednesday to give a result or – you know what I mean – if they're not there when the result comes back, how does, that sort of flicks onto somebody else's workload and that's a really bad thing in terms of – but it doesn't always work both ways. So in other words people who work more FTE tend to send more patients and then obviously they're available for most of their own patients, but they're also going to be in getting other people's patients as well, and with acute care there's no wriggle room CG10	Resource intensive program with workforce who largely work part time
I actually found it very straight forward, but then that may be – we also have here, or had here, [project officer name] who was involved and [genetic counsellor name] one of our genetic counsellors who had quite a lot of involvement. CG11	Provision of resources facilitated engagement with the program

By Phase

**Pre implementation**
CFIR construct: relative advantage
Definition in context: Stakeholders' perception of the advantage of implementing UR genomics in acute care versus an alternative solution.

Unfortunately, I didn't get an answer with either of my babies, but regardless of that I think knowing that they've had a full exome – clinical exome within 24, 48 hours was really helpful just to be able to show the parents we didn't find anything, we had a good look, that's actually quite helpful for people I think. CG11	Perceived benefit for parents despite no results
I think the hope is that we move away from a diagnostic process which before was more of within research setting, to something that can be a new thing in the clinical setting, and as well to increase familiarity with it. I4	Bringing practice into everyday clinical use
I do think the results were very useful to the families but I think they would have been just as useful if they'd arrived two weeks later CG6	Not sure of the advantage of speed
trying to get a bit more detailed information, ah, and getting that information, ah, in a quicker time frame, ah, so that we can, ah, guide the management accordingly, ah, that was the hope. I5	Perceived advantage of speed of turnaround on patient care
we wanted to have a pathway that would give us access to faster results for the kids in ICU, because up until – so things are changing so rapidly that even what we were thinking a year and a half ago seems really out voted now, but before now, 18 months ago, it was really different. If we had a kid in ICU and we thought they were very sick and we thought they had Charge syndrome, we'd organise a Charge syndrome test – maybe targeted. And we wouldn't be surprised if that took us 3 weeks to get a result, and we would try to do that through whatever pathway we could, whether that was overseas or local. So we had – we had a very – much longer – tolerance of, much longer times to get results. But with this project we saw it as an opportunity to get more rapid results, which we thought would be beneficial for many of the families that we were seeing. CG10	Speed of testing in this program perceived to be of benefit to families
CFIR construct: design packaging and quality	
Definition in context: Perception of how UR genomics in acute care is presented and assembled.	
And, I guess, sorry, other concern about the impact on service provision and how we were going to deal with it because we're not really used to dealing with. After hours particularly and the fact that you have to compress what you'd normally do over a long period of time into a short period of time and potentially volume, load of how many cases we were going to get. CG8	Concern about set up of the program (ties to resources)
I think it was a well-structured, rigorous project. CG4	Design of the program
I think make sure you get yourself rostered on with an excellent genetic counsellor because they will help you enormously. CG8	Improved quality of service (ties to resources)

think getting the timing right is tricky for some patients, because we kind of sometimes – like, this patient, for example, that I saw who was referred to the genetics service and, I guess, when we saw them, as a consult, considering what genetic testing, if any, was worthwhile, it was on the day that they'd had serious cardiac surgery and were in the intensive care unit. And, so, it was difficult CGT4	Caution about the timing of delivering offer to families
My understanding is they [NICU] like the multi-disciplinary team case conference with the patient, with the family type of model. So, you know, we'd be happy to work with them, I suppose, to continue to do it that way if that's what they wanted. CG2	Program design to engage multiple disciplines (tie to networking)
**Post implementation**	
CFIR construct: networks and communication	
Definition in context: Nature and quality of i) social networks and ii) formal and informal communications within either the home organisation or the acute care team	
Things that have worked very well. I mean we have a very streamlined communication system in our team whereby the fellow and/or consultant who see the patient then put it to the committee and then obviously the lead, she puts the call out. And the callout is to our genetic counselling team actually. So that's a call out for someone to do the consent process or whatever. Once that happens, the communication is via our Acute Care genomics email thread as well. GC2	Process of communication
I think because we're building upon, standing on the shoulders of giants so to speak, we're building upon what we've already done so we've built these systems and many relationships especially with the different consultants involved GC3	Benefits of previous networking and relationship building
Well, I think us being up there, seeing the patients, doing the first few, talking to our colleagues there and saying, "This is what we're looking for. This case that you've referred us isn't quite right for these reasons," or, "This one is perfect. That's exactly what we're looking for," and things like that. So, those, kinds of, discussions which we probably wouldn't have had with them previously about exomes because, you know, who's got time to go and talk to every single consultant in a hospital about what you're looking for in an exome, but that project definitely put us together and yeah. I think the neonatal intensive care consultants now have a very good idea of what an exome can and can't do and what kinds of patients to select for that, whereas previously they didn't. But it did take them time to learn that and, I guess, that's, because they're not just one person. There's a team and also, it takes you a little while to shift your thinking about things to where it ought to be. CG2	Engaging intensivists – building relationships

I just don't know if the other geneticists in my department didn't push it, because we all see many consults daily, and so I think that was frustrating. Apart from myself, one of the other geneticists would often put it through, it just wasn't advertised well enough in the beginning, that's what I feel. Yeah. CG7	Communication about the program in the early stages
If we would know that once per week [the] genetic team comes around we could actually batch questions to them and then discuss the case, and that may just help to build up that relationship. At the moment it is still there but ad hoc, and it depends a little bit of that level of suspicion from within ICU team, or the genetics team, as to when a geneticist gets involved I4	Process and relationship building between genetics and intensivists
CFIR construct: design packaging and quality	
Definition in context: Perception of how UR genomics in acute care is presented and assembled.	
one thing that was a little bit laborious and you could see that the parents became quite frustrated was the amount of consent forms and I also think that parents just wanted to spend time with their babies and they didn't want to have to listen to pages and pages and pages of medical jargon GC5	Caution over the way consent was presented
Personally for me I think it worked – I mean, apart from not getting answers as you just said but no, I thought the idea of it… you know, one of our two babies, one of the two babies that I had, parents were from somewhere, Afghanistan or something like that I think, anyway, non-English speaking, so consent for something complex like this was maybe the only issue. CG11	Program well designed – still concern over length of consent process
The last thing is that the lab process needs to be modelled on the way [Organisation name] does it. CG7	Potential improvements to the way processes can be assembled
what we realise with this project is when we rang the genetic consultant we got actually a response very quickly, and we got an assessment of the genotype very quickly. So, in a way it seemed to have increased as well the interest of the genetic service in the ICU patients, and the other key message for intensivist is that the intensivists does not need to make an assessment on the likelihood whether this is genetic or not, and what's the most appropriate way of diagnostics that's being taken over by the genetics team. I4	Program design engaged genetic staff and intensivists
**Future**	
CFIR construct: networks and communication	
Definition in context: Nature and quality of i) social networks and ii) formal and informal communications within either the home organisation or the acute care team	

The other thing I thought about is the actual variant curation and being part of those – I didn't get a chance to be part of all of them but just a couple of them. I really valued and even though – it actually went very quickly and they were just sort of more into, like, yes/no, yes/no, blah, blah, blah, it was still interesting to see how they do it. CGT3	Engagement with the process
I would say, you know, it's very important to have very clear dialogue with the NICU consultants and the PICU consultants. So, that would be one thing. And then working well as a team and utilising your genetic counsellors well, and your clinical geneticist well for the things that they do, and not, kind of, wasting time trying to do each other's jobs. That is a very helpful thing. We got our genetic counsellors to help out with the consenting part of the recruitment and the clinical geneticist fellows did the, I guess, diagnostics side and the test ordering. GC2	Communicating clarity around roles
The other thing I liked was the jury system [virtual intake meetings]. Do you know about that? How we decided - I'm on that panel where we would decide - a case would be put up for approval and as long as we got a critical mass of people saying, yes, we thought it was an appropriate case, it got approved; I thought that was very collaborative, and there's about seven or eight of us that are on that committee that have approved or not approved cases.CG5	Building relationships (ties to design quality)
Communication was wonderful, I loved the email even though sometimes it can be going off in the background, you know, one email, two emails, eight emails later but I do like that, kind of, everyone's included in the communication because it just helps everyone know where things are at for that patient and I think that's really important and everyone's aware of what's going on. GC7	Process and benefits of communication
CFIR construct: Available resources	
Definition in context: Resources dedicated for implementation of ultra rapid genomics and on-going operations, including money, training, education, physical space, and time.	
So, I think equity of access is another thing that is something that I've definitely been thinking about. In fact, I'll give you an example. One of the babies who wasn't eligible, we ended up doing an urgent clinical exome for them anyway. So six-week turnaround time, they have a life-limiting X-linked condition. And so for me, that's probably the biggest thing for me, is the equity of access. So, if we're going to offer this, we need to be offering it through the state. GC6	Impact of lack of resource
I don't see intensivists or any neontologists to have the expertise to say, "These are the right patients to select." So, you would still need a filtering process where the geneticist checks the phenotype against the possible genotype, then say, "Okay, this is worth investing," because it's still, as I said, a fairly expensive undertaking obviously. I4	Need for genetic staff
So, I think, from a time and resourcing perspective, you know, as long as you plan for it you can do it. CG2	Planning helps mitigate shortfall in resources

I guess, the first thing I would say to them is it takes a lot more resources than you can possibly imagine. Plan for that really well. You're going to need a lot more than you think you need. So, that would be the number one thing. I was quite surprised at how labour intensive it is. GC2	Resource intensity
*We've had a couple of genetic counsellor get-togethers, not just us, but with the other GCs around Australia. We've had two or three sessions where we've talked about what's worked well and what hasn't and presented cases. And I think here, we've decided that the doctors talking to their peers works better than one of us standing there saying, come on.*GC1	Allocation of resources (ties to communication)

By Role

**Clinical Geneticists**	
CFIR construct: design packaging and quality	
Definition in context: Perception of how ultra rapid genomics in acute care is presented and assembled.	
I think it's a hugely ambitious thing to develop and deliver and I thought it was delivered – it was really high calibre processing of the tests, high commitment from the clinical and laboratory teams to iron out problems as they arose or get a system that works. CG4	Quality of program
I think that the genetic counsellor needs to – whoever is doing the ultra-rapid exome consent etc and talking to the family to - that person needs to have – to be on call for a week and then off for a week, there needs to be a time when that person is not on call for acute care exome. I think there should always be a geneticist involved, I think there needs to be at the [Hospital 4] site a better engagement of the CICU, the NICU staff, and involving a ward round with a geneticist weekly, whoever the lead geneticist is for the site. And the last thing is that the lab process needs to be modelled on the way [Organisation name] does it. CG7	Models of service delivery
there's a whole lot of cases that I felt I wouldn't think are good cases for the ultra-flagship because I didn't think the clinical scenario was one that really warranted everyone's time and effort, and I know that wasn't necessarily the idea, but I couldn't help just think it was wasting everyone's time when for the sake of a few days, or a little bit more clinical time, the scenario changed a little bit and actually the need for the ultra-exome, or whatever, wasn't so important. CG9	Query around design of rapid turnaround
I think it's a hugely ambitious thing to deliver and I thought it was delivered – it was really high calibre processing of the tests, high commitment from the clinical and laboratory teams to iron out problems as they arose or get a system that works. CG4	Quality of program

Genetic Counselors	
CFIR construct: executing	
Definition in context: The process of implementing ultra rapid genomics in line with the plan	
So because they don't have an electronic medical record, the child's request can be sent through the normal system so to speak, on the ward, or put into the computer and it pops up on the bed nurses' screen as something they need to do and they get to it when they get to it or you politely ask them to hurry it along, yeah, from the – at the beginning we were manually carrying the parental bloods back and so I would take the path slip and stand there and wait and bring it down to the laboratory but slowly they've been putting systems in place to automate that a little bit more so that we're not having that situation of genetic counsellors running, quite literally, running bloods down the hall and getting them to where they need to be. GC3	Establishing processes
now that we've got the pathways set up it's much easier but there were those teething issues about do we need blood or are we just taking DNA? And, okay, now we're using blood so we have to re-collect and who's going to collect blood from the parents and all of that stuff. GC7	Establishing processes
How are we delivering them from our site to the site that are actually sending the sample, what paperwork needs to be filled out. I know that [genetic counsellor name] had a lot of kerfuffle about, okay who exactly needs to fill out what consent form and where. There was a little bit of uncertainty, again about it, sort of getting teething problems about how exactly are the parents being tested. GC6	Establishing processes
You have a changing team there, so sometimes you need to go in and explain. I usually, when we go and do a consent, it's usually a genetics fellow or a consultant who will go with the genetic counsellor. And these days I will ask the doctor to go and talk to the other doctors and sort out this blood business while I do the consent with the parents. GC1	Establishing processes
**Intensivists**	
CFIR construct: access to knowledge	
Definition in context: Ease of access to digestible information and knowledge about UR genomics in acute care and how to incorporate it into work tasks.	
I think here the clinical geneticists have made a big made a big difference but somethings is just that personal level, because if you comfortable to ring someone you know, and I think the way the process works is that once genetics is involved all the gate control, or it's the genetics team which I think it's obvious then because they have the biggest expertise to make sure the test is not used excessively. I4	Engaging with the clinical geneticists
if we would know that once per week genetic team comes around we could actually batch questions to them and then discuss the case, and that may just help to build up that relationship. At the moment it is still there but ad hoc I3	Setting up regular sessions with genetics

	(links to networking)
[Program lead had] given us at the consultant level – when she was starting to, wanting to roll this out – she'd given us a talk and a tute [tutorial]. [Genetic counsellor name] also gave us a tute [tutorial] about realising what exomes mean and what information you can get, what information you can't get, so we got very well educated at the consultant level so as I said [program lead] and [genetic counselor] both, I felt that we got a good education. I1	Benefit of early knowledge sharing
So, I think it's just the genomics team enable people to very easily adopt it into practice I2	Impact of genetic staff sharing knowledge
Any questions that we had with the results were explained by our geneticist. We don't just get dumped with the result, the result gets explained to us so that then we can explain it to the parents so, no, I think we're lucky because as I said we have a very close relationship with our genetic colleagues and they will always come and help us deliver news if we don't understand what we're delivering. I1	Benefit of engagement with clinical geneticists (ties to networking)
